# Development of a topic‐specific bibliographic database supporting the updates of SPIRIT 2013 and CONSORT 2010

**DOI:** 10.1002/cesm.12057

**Published:** 2024-05-15

**Authors:** Lasse Østengaard, Ariel Barrientos, Isabelle Boutron, An‐Wen Chan, Gary Collins, Sally Hopewell, David Moher, Camilla Hansen Nejstgaard, Kenneth F. Schulz, Benjamin Speich, Evan Tang, Ruth Tunn, Nozomi Watanabe, Chenchen Xu, Asbjørn Hróbjartsson

**Affiliations:** ^1^ Department of Clinical Research, Centre for Evidence‐Based Medicine Odense (CEBMO) and Cochrane Denmark University of Southern Denmark Odense Denmark; ^2^ Open Patient data Explorative Network (OPEN) Odense University Hospital Odense Denmark; ^3^ University Library of Southern Denmark Odense Denmark; ^4^ Temerty School of Medicine University of Toronto Toronto Ontario Canada; ^5^ Centre for Research in Epidemiology and Statistics (CRESS) Université Paris Cité, Université Sorbonne Paris Nord, Inserm, INRAE Paris France; ^6^ Cochrane France Paris France; ^7^ Department of Medicine, Women's College Research Institute University of Toronto Toronto Ontario Canada; ^8^ Centre for Statistics in Medicine University of Oxford Oxford UK; ^9^ Oxford Clinical Trials Research Unit/Centre for Statistics in Medicine University of Oxford Oxford UK; ^10^ Clinical Epidemiology Program Centre for Journalology, Ottawa Hospital Research Institute Ottawa Ontario Canada; ^11^ School of Epidemiology and Public Health, Faculty of Medicine University of Ottawa Ottawa Ontario Canada; ^12^ Department of Obstetrics and Gynecology, School of Medicine The University of North Carolina at Chapel Hill Chapel Hill North Carolina USA; ^13^ Department of Clinical Research, CLEAR‐Methods Center, Division of Clinical Epidemiology, University Hospital Basel University of Basel Basel Switzerland; ^14^ Faculty of Medicine University of Toronto Toronto Ontario Canada; ^15^ Cardiovascular European Research Center (CERC) Massy France; ^16^ Faculty of Medicine University of Ottawa Ottawa Ontario Canada

**Keywords:** bibliographic database, CONSORT (CONsolidated Standards Of Reporting Trials), reporting guidelines, SPIRIT (Standard Protocol Items: Recommendations for Interventional Trials)

## Abstract

**Introduction:**

An important mechanism of research waste is inadequate incorporation of, and references to, previous relevant research. Identifying references for a research manuscript can be challenging, in part due to the exponential rise in potentially relevant literature to consider. For large research projects, such as developing or updating reporting guidelines, it may be helpful to construct a supportive topic‐specific bibliographic database.

**Methods:**

In support of updating the Standard Protocol Items: Recommendations for Interventional Trials (SPIRIT) 2013 and the CONsolidated Standards Of Reporting Trials (CONSORT) 2010, we developed the SPIRIT‐CONSORT Evidence Bibliographic database (SCEBdb): a freely available topic‐specific bibliographic database of publications providing an evidence foundation for the updates. We searched multiple sources of potential publications and tagged included ones with database‐specific keywords. For context, we also formulated 10 core considerations for constructing topic‐specific bibliographic databases and identified and described 5 illustrative other databases.

**Results:**

As of April 2024, the SCEBdb included 846 publications. The database proved useful as a supplementary information source for our scoping review of published comments on SPIRIT 2013 and CONSORT 2010, for a supplementary Delphi process, and in the writing phase of the guidance documents. We expect that the database will be useful for future projects within the fields of clinical research methodology, bias, evidence synthesis, and randomized trials.

**Conclusion:**

The methods involved in constructing the SCEBdb, and our suggested core considerations for topic‐specific bibliographic databases, could be helpful for researchers reflecting on whether, and how, to develop a topic‐specific bibliographic database.

## 1 INTRODUCTION

Across all academic fields, it is common practice for authors of scientific publications to include references to previous studies. In the ideal scenario, references are identified and selected on academic merit to communicate, in a balanced manner, an explicit and conscientious link between the new study and the relevant scientific literature.

Identification and discussion of previous studies (e.g., related to epidemiological background, estimations of intervention effects, or theoretical considerations), provides the basis for framing the relevance of the research question posed by the new study. It also supports reflections on what the new study adds to the results of previous ones, whether the chosen methods were adequate, and more generally how the pattern of results relates to what is already known. Ideally, references communicate a web of pre‐study theoretical and empirical evidence, tailored specifically for the study at hand.

However, the overwhelming and increasing number of scientific publications [1] challenges this ideal, and lack of references to, and awareness of previous core studies is both frequent and a central mechanism of research waste. [2, 3] This has prompted calls for a more systematic approach to referencing in general, and for no new studies to be planned without a preparatory systematic review. [4] In the extreme case, many important existing studies may be overlooked. [5, 6] For example, Fergusson et al. [6] analyzed 64 randomized clinical trials of aprotinin to limit bleeding in cardiac surgery published between 1987 and 2002 and found that the majority of trials did not adequately cite the previously published studies. After the twelfth trial, an effect of aprotinin was established, yet a further 52 trials were conducted.

Researchers can choose a variety of strategies to seek to identify all relevant previous studies. One pragmatic but risky option for researchers is to trust that their background knowledge of the field suffices. Another option is to search a single database intermittently and informally, whereas a third strategy is to conduct a formal search in one or more databases. However, in large or complicated research projects, it may be relevant to construct a formal topic‐specific bibliographic database.

An example of such a large project is developing and updating reporting guidelines. Reporting guidelines address multiple aspects of a given type of research publication, and need regular updating due to evolving research practices, methodological innovations and changed reporting and publication practices.

As of April 2024, several of us are finalizing the update of the SPIRIT 2013 and CONSORT 2010 reporting guidelines. [7] SPIRIT guides the reporting of trial protocols and was published in 2013. [8] CONSORT guides the reporting of completed randomized trials, and was first published in 1996, [9] updated in 2001 [10] and again in 2010. [11] Our current updating approach involved a scoping review of published comments on SPIRIT 2013 and CONSORT 2010, [12] a Delphi process, [13] a virtual consensus meeting, a face‐to‐face meeting, and the formulation of the updated checklists and accompanying Explanation and Elaboration (E&E) documents.

To support this process, we developed the SPIRIT‐CONSORT Evidence Bibliographic database (SCEBdb): a topic‐specific bibliographic database of publications providing an evidence foundation for the updates. In this paper, we describe the development of the SCEBdb, with an emphasis on procedures that could be useful for researchers reflecting on whether and how to develop topic‐specific bibliographic databases. For context, we also formulated 10 core considerations for constructing topic‐specific bibliographic databases and identified and described 5 illustrative examples of other databases.

## 2 METHODOLOGY

### 2.1 Terminology

We use the term “topic‐specific bibliographic database” for databases that select publications on a specific scientific problem, field, or publication type. Major bibliographic databases, such as MEDLINE and Embase, are not topic‐specific as they select publications by journals.

### 2.2 Eligibility criteria

We included publications relevant to the updates of the SPIRIT 2013 and CONSORT 2010. The eligibility criteria are elaborated in Table 1.

**Table 1:** Ten core considerations when deciding whether and how to develop a topic‐specific bibliographic database.

	Core general considerations	Core general considerations applied to the SCEBdb
Aim	The aim of a topic‐specific bibliographic database is the single most important thing to consider. One way of formulating the aim is by specifying the database's topic, the type of publications intended to be included, and the intended operational complexity (e.g., database‐specific keywords and updates). A bibliographic database with a broad aim could support a large research project, for example, a reporting guideline. A database with a narrower aim could support a sizeable research project. For many other scientific projects, it would be rational for researchers to decide against constructing a formal database.	The aim of the SCEBdb is to provide an evidence foundation for the updates of SPIRIT 2013 and CONSORT 2010 reporting guidelines. This aim is broad because the SPIRIT 2013 and CONSORT 2010 reporting guidelines address multiple aspects of the reporting of protocols for clinical trials and the reporting of completed clinical trials, and many publications are therefore relevant to the database.
Type of publications	Another important choice is to define what type of publications on the topic the database should strive to include (eligibility criteria). Highly interesting publications will often be relatively easy to define, but the challenge is to define the threshold for gray‐zone publications (of limited or potential topical interest) or publications potentially of interest to future projects.	Our eligibility criteria were: (1) empirical studies of SPIRIT 2013 and CONSORT 2010 of any kind (e.g., studies investigating adherence to SPIRIT 2013 or CONSORT 2010); (2) studies comparing reporting of protocols with published randomized trials; (3) meta‐epidemiological studies of bias in randomized trials; (4) other methodological studies of risk of bias and reporting relevant for items included in SPIRIT 2013 or CONSORT 2010 and; (5) other relevant publications for the update of the SPIRIT 2013 and CONSORT 2010 (e.g., commentaries suggesting modifications for one or both of the reporting guidelines). Gray‐zone publications were discussed (e.g., empirical studies on SPIRIT‐CONSORT extensions), and the project coordinator wrote out to the involved persons how to deal with them. The topic of interest for the SCEBdb was publications relevant to the update of the SPIRIT 2013 and CONSORT 2010 reporting guidelines.
Topic and keywords	The database's topic of interest is the scientific field (e.g., “colorectal cancer”) or study type (e.g., randomized trial) that it addresses. The formulation of the topic of interest can be broad (e.g., “cancer”) or narrow (e.g., a specific type of cancer or cancer in a specific region), and this decision will have a considerable impact on the utility and necessary resources for the database. A crucial element in developing a database is how the publications are organized. Providing publications with relevant database‐specific keywords is crucial for the usefulness of the database.	We categorized the included publications using database‐specific keywords (i.e., tags). Each publication in the SCEBdb was provided with at least one keyword. The keywords were given to indicate; (1) which item(s) in the SPIRIT and/or CONSORT reporting guidelines (there were 46 different items) the publication is relevant for; (2) that the publication is relevant for multiple items in the SPIRIT or CONSORT reporting guidelines; (3) that the publication is an empirical study of bias; (4) that the publication provides a comment suggesting modifications to the SPIRIT and/or CONSORT reporting guidelines and; (5) that the reference is deemed relevant but does not fit into the above‐mentioned categories.
Resources	Constructing a formal bibliographic database is resource demanding, and the need for resources increases with a broadly defined aim. The main factors impacting on resources are number of titles/abstracts screened (inclusiveness of which publications are regarded relevant, and sensitivity of search), use of two screeners, reading and adding keywords to full texts, and updating.	With the broad aim of the SCEBdb, we knew that the development of the database would be time‐consuming. Therefore, we prioritized only having one screener in the title abstract screening, and for the updating of the database, even though having two screeners would have reduced further the small risk for overlooking more than a few relevant publications.
Coordination	Developing a bibliographic database will often require a considerable degree of coordination and includes several persons to screen the references and the full‐text publications. Having one designated project coordinator, and guiding documents, facilitates comparable practices between persons.	A research librarian (L. Ø.) was the designated project coordinator for the development of the SCEBdb. First, we developed a document describing the database (Supporting Information: Appendix 1), but we found it relevant to develop two further guiding documents to help the screeners during the screening procedures to facilitate the most optimal practice (Supporting Information: Appendices 2 and 3).
Search	Once the criteria for eligible publications have been defined, the next step is to decide the balance between sensitivity and specificity in the search process to identify such publications. A very sensitive search (including multiple major databases and very sensitive search terms) can take too much time, and a too specific search may miss relevant publications. We suggest an iterative approach where the search strategy is continuously refined and supplemented by an informal approach, for example, personal files.	Because the SCEBdb has a broad aim, we needed to be careful when we developed the search strategy. We searched for the first four elements mentioned under “Type of publications,” and the last element was identified through our supplementary searches. We decided to have a dynamic search strategy which could be adjusted when necessary.
Software	There are numerous software options for managing and displaying the database. A simple approach is to present the database in an Excel sheet, and a more advanced approach is to present the database on a website. If the database is intended for a small number of users, a simple approach is recommended. But if the database is intended for many users, we recommend displaying the database on a user‐friendly website (i.e., with easy access and simple search features).	Zotero was chosen as the software to host the SCEBdb, enabling collaborators to work together from different countries and to easily access the notes and keywords provided by all. Many researchers use the same or similar software tool to manage their references so the user interface is familiar to many and intuitive. We decided to postpone the decision of displaying the SCEBdb on a website until after the reporting guidelines had been updated. If the interest from the methods research community is sufficient, we plan to switch to a web‐based platform.
Access	A database can be made accessible in different ways. A database can be stored at a research center, and external users may request access. A database can also be stored on a website that requires log‐in, and finally, a database can also be freely accessible. The accessibility of the database is an important choice because it will affect the user‐friendliness of the database.	The SCEBdb is accessible via Zotero. This enables others, who find the topic of the database interesting, to access the database in an easy way. Users of the SCEBdb do not need to install any software because they can access the database via the online version of Zotero, https://www.zotero.org/groups/4548554/the_scebdb/library. This minimizes the difficulties with accessing the database.
Updating	If the database is aimed at supporting future research projects, it will often involve continuous updates. It is important to consider the timing, logistics, and resources necessary for the updates. We suggest that an update every 3–12 months will be adequate for most databases.	The searches in Medline (Ovid) and Embase (Ovid) are updated monthly. We decided that frequent update of the SCEBdb was important until the guidelines had been finalized.
Evaluation	It is relevant to consider re‐evaluating the procedures involved in the database, both during initial construction and after some time in use. The most relevant aspects to consider are the continued relevance of the database, the adequacy of the eligibility criteria, the search strategy, the degree of user‐friendliness, and the possibility of simplifying procedures, for example, frequency of updates.	Throughout the development of the SCEBdb, L. Ø. and A. H. evaluated different elements, for example, the adequacy of the search, and the logistics of multiple screeners. We expect that the frequency of updates be reduced to four times per year when the SPIRIT 2013 and CONSORT 2010 reporting guidelines have been updated. L. Ø. will be responsible for updating the database. The classification system will most likely also be revised because new elements will be included in the reporting guidelines.

Abbreviations: CONSORT, CONsolidated Standards Of Reporting Trials; SCEBdb, SPIRIT‐CONSORT Evidence Bibliographic database; SPIRIT, Standard Protocol Items: Recommendations for Interventional Trials.

### 2.3 Search strategy

We searched MEDLINE (Ovid) and Embase (Ovid) and supplemented with searches in Web of Science, the group members' personal files, and any suggestions from other interested parties (e.g., authors who suggest their own papers).

We limited our search to publications from 2010 (last version of CONSORT). The initial searches were conducted in June 2020 (and updated monthly since).

### 2.4 Screening and organization of the references

First, the unique records (references, titles, and abstracts) were screened for eligibility by a single person. The references were excluded if they, for obvious reasons, were not relevant (e.g., publications that only had used SPIRIT or CONSORT as a reporting guideline).

Second, full‐text documents were screened by a single person. The relevant publications were provided with short notes, for example, “CONSORT: Item 13, participant flow.”

Third, each included publication was read by a single person from the SPIRIT‐CONSORT executive group, double‐checking its relevance. That person also categorized included publications with database‐specific keywords (i.e., tags) linking a publication to specific reporting items in both guidelines (Table 1).

### 2.5 Software

We used Covidence (www.covidence.org) for the screening procedure and Zotero (www.zotero.org) as reference manager software to host the database.

### 2.6 Updating

A single person screened newly identified references once a month. We plan to continue this until the guidelines have been finalized.

### 2.7 Resources

To evaluate the use of resources, we retrospectively asked the involved persons how much time they have spent on the project.

### 2.8 Usefulness

In November 2023, the SPIRIT‐CONSORT executive group (*n* = 7) was asked about their experiences with the database while writing the updated SPIRIT and CONSORT reporting guidelines.

### 2.9 Core search functions in the SCEBdb

The SCEBdb has two main search functions. The first is the search function in Zotero, for example, searching for terms in the title/abstract. The second option is to filter the search using the database‐specific keywords (i.e., “tags” in Zotero). For example, the majority of the keywords in the SCEBdb link to specific SPIRIT or CONSORT item(s).

### 2.10 Availability

The database can be accessed in Zotero: https://www.zotero.org/groups/4548554/the_scebdb/library.

### 2.11 Other topic‐specific databases and core considerations

We searched for other topic‐specific databases in PubMed, Open Science Framework, Zotero, and Google Scholar, and selected five illustrative examples for description. We discussed iteratively the core considerations involved in developing a topic‐specific bibliographic database.

### 2.12 Additional information

For additional information about the methodology see Supporting Information: Appendices 1–3.

For context, we have displayed the differences between a scoping review and a topic‐specific database in Table 2.

**Table 2:** Differences between a scoping review and a topic‐specific database.^a^

	Scoping review	Topic‐specific database
Type of aim	The aim of the scoping review was focused.	The aim was broad and many publications with different focuses were relevant.
Search	Sensitive and comprehensive.	Less sensitive than the scoping review. Comprehensive but with no claim of completeness.
Update	Non. The searches were conducted one time.	The searches have been updated monthly since June 2020.
Literature management	The tools for literature management played a minor role. Duplicates were removed using EndNote, and the screening was done in Covidence	The tools for literature management played a prominent role because the database is displayed in Zotero. Deduplication and the screening were made in Covidence.
Synthesis	The results were analyzed and summarized.	Non. There was no formal synthesis of the references in SCEBdb.
Usefulness in the project	Gave a thorough overview of the comments and suggestions for modifications on SPIRIT 2013 and CONSORT 2010, which was used to inform the Delphi survey.	It was useful in many different stages (i.e., the scoping review, the Delphi survey, and the writing phase) of the update of the SPIRIT 2013 and CONSORT 2010 reporting guidelines.

Abbreviations: CONSORT, CONsolidated Standards Of Reporting Trials; SCEBdb, SPIRIT‐CONSORT Evidence Bibliographic database; SPIRIT, Standard Protocol Items: Recommendations for Interventional Trials.

^a^ We have displayed the differences between the scoping review of published comments on SPIRIT 2013 and CONSORT 2010 [12] and the SCEBdb.

## 3 RESULTS

### 3.1 SCEBdb

**Figure 1:**
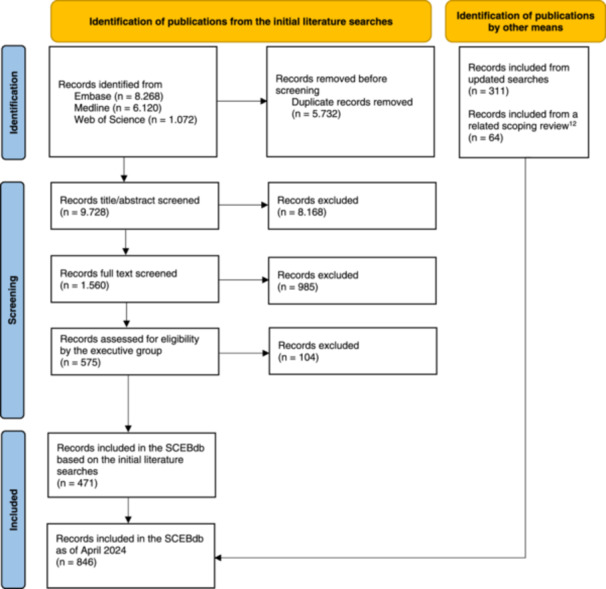
Flow diagram. [13]

From the initial search, 9728 unique records were identified, and as of April 2024, the database included 846 publications (Figure 1).

The database was used as a supplementary source of publications to include in the scoping review of published comments on SPIRIT 2013 and CONSORT 2010. [12] The scoping review was primarily based on its own search strategy specifically aimed for comments, identifying 79 eligible publications, but the SCEBdb identified 14 additional publications. The SCEBdb was also useful during the development of the Delphi survey. [14] However, the database proved especially valuable in the writing phase of the guideline documents as a means to quickly locate and access relevant publications. All executive group members expressed that it was helpful and used phrases like “I found it incredibly helpful…,” and “this has been a great resource….”

It took a total of 80 working days for 15 people to finish the initial title/abstract screening (approximately 20 working days), the full‐text screening (40 days) and the final screening by the executive group (20 days), that is, approximately 1 week's work per person, on average. In addition, the coordinator used approximately 35 days for planning, coordination, logistical support, and updating.

### 3.2 Other topic‐specific databases and core considerations

In Table 1, we formulated 10 general core considerations for constructing topic‐specific bibliographic databases and applied these to the SCEBdb. We characterized five examples of topic‐specific databases in Table 3 and listed others in Supporting Information: Appendix 4. [15, 16, 17, 18, 19]

**Table 3:** Five examples of publicly available topic‐specific bibliographic databases.^a^

Title	Topic	Publications: *n*, year‐span, and source	Search feature and update	Accessibility
Smartphone‐RCCT [15]	Randomized clinical trials of smartphone applications for chronic condition	*n* = 73 Publication years: 2011–2019 Source of publications: MEDLINE, Web of Science, PsycInfo, and CINAHL. The search strategy may be improved in future updates.	Search feature: Search by keywords in the title. The results can be filtered by different categories, for example, study design, setting, participants, and outcomes. Updated: Annually.	Accessibility: A text file and Excel spreadsheet of the database can freely be downloaded. Link: https://www.osf.io/nxerf/
The Diet‐Related Fibers and Human Health Outcomes Database [16]	Fiber and physiological health	*n* = 1.156 Publication years: 1966–2017 Source of publications: MEDLINE.	Search feature: Search by keywords. Updated: No description of the current situation. Annual updates were planned through 2017.	Accessibility: An Excel spreadsheet of the database can freely be downloaded on an online repository. A log‐in to the repository is required. Link: https://srdrplus.ahrq.gov/ (search for *Diet‐Related Fibers and Human Health Outcomes, Version 5.0*)
The Justice Database [17]	Publications on philosophy of justice	*n* = 1.220 Publication years: 1923–2021 Source of publications: Not stated.	Search feature: Search by keywords. The publications are organized in folders related to schools of justice philosophy (e.g., libertarianism, feminism) and applied approaches (e.g., environmental, energy). Updated: “living resource” (search frequency NA).	Accessibility: The database is freely be accessed in the reference manager software Zotero and be downloaded as an Excel file. Link: http://zotero.org/groups/4662062/justnorth_justice_database/library
The ORRCA database [18]	Recruitment research in clinical trials	*n* = 4.813 Publication years: 1976–2020 Source of publications: Multiple sources incl. MEDLINE, Scopus and Cochrane Database of Systematic Reviews.	Search feature: Search by keywords in, for example, the title and abstract. Selection of different recruitment domains can filter the results. Updated: Annually.	Accessibility: Can freely be accessed on a website, and Excel spreadsheets can be downloaded with data. Link: https://www.orrca.org.uk/
The PTSD‐Repository [19]	Randomized clinical trials for posttraumatic stress disorder	*n* = 496 Publication years: 1988–2023 Source of publications: PTSDpubs, MEDLINE, PsycINFO, Cochrane CENTRAL, Embase, CINAHL, and Scopus.	Search feature: Search by keywords. The results can be filtered in Excel by different categories, for example, military status (e.g., active duty and veteran), intervention, PTSD severity, and risk of bias rating. Updated: Annually.	Accessibility: Can freely be accessed on a website, and Excel spreadsheets can be downloaded with data. Link: https://ptsd-va.data.socrata.com/

^a^ The five examples are selected to show different types of databases based on the topic, size, and search feature.

## 4 DISCUSSION AND CONCLUSION

A main barrier for developing a topic‐specific bibliographic database is limited human resources. For the SCEBdb, 15 people each invested a week's work, on average. The workload of the coordinator was high, approximately 7 weeks. The time used was assessed retrospectively and should be interpreted cautiously. A minimal approach involving a repository style database without database‐specific keywords could probably be completed using much fewer working days.

A limitation of our approach is that we might have missed relevant publications because we tried to limit the use of resources for developing the SCEBdb. Missed publications which are later identified, will be added to the SCEBdb, and the search strategy will be revised.

The SCEBdb was a core resource for the authors of the SPIRIT 2024 and CONSORT 2024 reporting guidelines. However, supplementary searches in standard bibliographic databases were still necessary to cover areas not included by the database, for example, when new items were added to the guidelines.

The EQUATOR Network [20] includes a library for health related reporting guidelines for different study designs. The LIGHTS [21] inventory, and the LATITUDES network [22] include complementary libraries of publications on methods guidance and on validity assessment tools of health research. These three libraries can be regarded as topic‐specific databases in a very broad sense, that provide health researchers with curated collections of publications on reporting, methods, and validity assessment.

We have not identified previous publications describing the general core considerations for constructing a topic‐specific bibliographic database, though aspects of the development of other topic‐specific databases have been published (Table 3, Supporting Information: Appendix 4).

The SCEBdb has proved to be valuable in the writing phase of the SPIRIT 2024 and CONSORT 2024 reporting guidelines, where the relevant publications for each item in both guidelines could easily be identified. Also, we anticipate the continuously updated SCEBdb could be useful for future projects within the fields of clinical research methodology, bias, research synthesis, and randomized trials.

In conclusion, the methods involved in constructing the SCEBdb, and our suggested core considerations for topic‐specific bibliographic databases, could be helpful for researchers reflecting on whether, and how, to develop a topic‐specific bibliographic database. We recommend that researchers involved in large or complicated research projects (e.g., updating reporting guidelines) consider constructing a formal topic‐specific bibliographic database.

## AUTHOR CONTRIBUTIONS

**Lasse Østengaard**: Conceptualization; formal analysis; investigation; methodology; writing—original draft; writing—review & editing. **Ariel Barrientos**: Formal analysis; investigation; writing—review & editing. **Isabelle Boutron**: Formal analysis; investigation; methodology; writing—review & editing. **An‐Wen Chan**: Formal analysis; investigation; methodology; writing—review & editing. **Gary Collins**: Formal analysis; investigation; writing—review & editing. **Sally Hopewell**: Formal analysis; investigation; methodology; writing—review & editing. **David Moher**: Formal analysis; investigation; methodology; writing—review & editing. **Camilla Hansen Nejstgaard**: Formal analysis; investigation; methodology; writing—review & editing. **Kenneth F. Schulz**: Formal analysis; investigation; methodology; writing—review & editing. **Benjamin Speich**: Formal analysis; investigation; writing—review & editing. **Evan Tang**: Formal analysis; investigation; writing—review & editing. **Ruth Tunn**: Formal analysis; investigation; writing—review & editing. **Nozomi Watanabe**: Formal analysis; investigation; writing—review & editing. **Chenchen Xu**: Formal analysis; investigation; writing—review & editing. **Asbjørn Hróbjartsson**: Conceptualization; formal analysis; investigation; methodology; writing—original draft; writing—review & editing.

## CONFLICTS OF INTEREST STATEMENT

B. S. received unrestricted grants from Moderna (2021/22) for studies unrelated to the presented work. I. B., A.‐W. C., G. C., S. H., D. M., K. F. S., R. T., and A. H. are part of the joint executive group for the planned updates of SPIRIT and CONSORT. The remaining authors declare no conflict of interest.

## PEER REVIEW

The peer review history for this article is available at https://www.webofscience.com/api/gateway/wos/peer-review/10.1002/cesm.12057.

## Supporting information

Supporting information.

## Data Availability

Data are publicly available.

## References

[1] Bornmann L , Haunschild R , Mutz R . Growth rates of modern science: a latent piecewise growth curve approach to model publication numbers from established and new literature databases. Humanit Soc Sci Commun. 2021;8(1):224. 10.1057/s41599-021-00903-w

[2] Chalmers I , Bracken MB , Djulbegovic B , et al. How to increase value and reduce waste when research priorities are set. Lancet. 2014;383(9912):156‐165. 10.1016/S0140-6736(13)62229-1 24411644

[3] Sawin VI , Robinson KA . Biased and inadequate citation of prior research in reports of cardiovascular trials is a continuing source of waste in research. J Clin Epidemiol. 2016;69:174‐178. 10.1016/j.jclinepi.2015.03.026 26086727

[4] Lund H , Brunnhuber K , Juhl C , et al. Towards evidence based research. BMJ. 2016;355:i5440. 10.1136/bmj.i5440 27797786

[5] Hrobjartsson A . Rapid response: wrong information on previous studies of the clinical use of placebo. BMJ. 2004;329:944. https://www.bmj.com/rapid-response/2011/10/30/wrong-information-previous-studies-clinical-use-placebo 15377572

[6] Fergusson D , Glass KC , Hutton B , Shapiro S . Randomized controlled trials of aprotinin in cardiac surgery: could clinical equipoise have stopped the bleeding? Clin Trials. 2005;2(3):218‐232. 10.1191/1740774505cn085oa 16279145

[7] Hopewell S , Boutron I , Chan AW , et al. An update to SPIRIT and CONSORT reporting guidelines to enhance transparency in randomized trials. Nat Med. 2022;28(9):1740‐1743. 10.1038/s41591-022-01989-8 36109642

[8] Chan AW , Tetzlaff JM , Altman DG , et al. SPIRIT 2013 statement: defining standard protocol items for clinical trials. Ann Intern Med. 2013;158(3):200‐207. 10.7326/0003-4819-158-3-201302050-00583 23295957 PMC5114123

[9] Begg C . Improving the quality of reporting of randomized controlled trials. the CONSORT statement. JAMA. 1996;276(8):637‐639. 10.1001/jama.276.8.637 8773637

[10] Moher D , Schulz KF , Altman DG , et al. The CONSORT statement: revised recommendations for improving the quality of reports of parallel‐group randomized trials. Ann Intern Med. 2001;134(8):657‐662. 10.7326/0003-4819-134-8-200104170-00011 11304106

[11] Schulz KF , Altman DG , Moher D , et al. CONSORT 2010 statement: updated guidelines for reporting parallel group randomised trials. BMJ. 2010;340:c332. 10.1136/bmj.c332 20332509 PMC2844940

[12] Nejstgaard CH , Boutron I , Chan AW , et al. A scoping review identifies multiple comments suggesting modifications to SPIRIT 2013 and CONSORT 2010. J Clin Epidemiol. 2023;155:48‐63. 10.1016/j.jclinepi.2023.01.003 36669708

[13] Page MJ , McKenzie JE , Bossuyt PM , et al. The PRISMA 2020 statement: an updated guideline for reporting systematic reviews. BMJ. 2021;372:n71. http://www.prisma-statement.org/ 33782057 10.1136/bmj.n71 PMC8005924

[14] Tunn R , Boutron I , Chan AW , et al. Methods used to develop the SPIRIT 2024 and CONSORT 2024 statements. J Clin Epidemiol. 2024;169:111309. 10.1016/j.jclinepi.2024.111309 38428538

[15] Barth J , Wang J , Lopez‐Alcalde J , et al. Smartphone‐RCCT: an online repository of randomized controlled clinical trials of smartphone applications for chronic conditions. Trials. 2022;23(1):909. 10.1186/s13063-022-06849-x 36303168 PMC9615349

[16] Livingston KA , Chung M , Sawicki CM , et al. Development of a publicly available, comprehensive database of fiber and health outcomes: rationale and methods. PLoS One. 2016;11(6):e0156961. 10.1371/journal.pone.0156961 27348733 PMC4922652

[17] JUSTNORTH . *Justice Database 2022*. Accessed December 20, 2022. https://justnorth.eu/outcome/database/

[18] Kearney A , Harman NL , Rosala‐Hallas A , et al. Development of an online resource for recruitment research in clinical trials to organise and map current literature. Clin Trials. 2018;15(6):533‐542. 10.1177/1740774518796156 30165760 PMC6236587

[19] O'Neil ME , Harik JM , McDonagh MS , et al. Development of the PTSD‐repository: a publicly available repository of randomized controlled trials for posttraumatic stress disorder. J Trauma Stress. 2020;33(4):410‐419. 10.1002/jts.22520 32667076

[20] The EQUATOR Network . Accessed May 25, 2022. https://www.equator-network.org/

[21] LIGHTS – Library of Guidance for Health Scientists . Accessed April 3, 2024. https://lights.science/

[22] LATITUDES Network . Accessed April 3, 2024. https://www.latitudes-network.org/

