# Supplementary material for: Development of a topic‐specific bibliographic database supporting the updates of SPIRIT 2013 and CONSORT 2010
Source: Cochrane Evid Synth Methods. 2024 May 15;2(5):e12057. doi: 10.1002/cesm.12057 (PMC11795946; doi:10.1002/cesm.12057)
Supplement: Supplementary file 1 — Supporting information. [file CESM-2-e12057-s001.pdf]

Appendices

Name of the database:
The SPIRIT-CONSORT Evidence Bibliographic database (SCEBdb) has previously been called the SPIRIT-CONSORT Informative Publications database (SCIPdb) in documents from 2020 (displayed in the appendices).
We renamed the database to SCEBdb in 2023 because we identified a new database about plants called SCIPdb.[1]

1) Priya P, Patil M, Pandey P, et al. Stress combinations and their interactions in plants database: a one-stop resource on combined stress responses in plants. Plant J 2023;116(4):1097-117. doi: 10.1111/tpj.16497

Appendix 1:
The SPIRIT-CONSORT Informative Publications database (SCIPdb): evidence foundation for SPIRIT 2021 and CONSORT 2021

Lasse Østengaard and Asbjørn Hróbjartsson on behalf of the SPIRIT-CONSORT executive group.
24.05.2020

Introduction
In this note, we describe the construction of the SPIRIT-CONSORT Informative Publications database, i.e. the SCIP-db.

We aim for a database of publications providing an evidence foundation for the planned 2021 updates of SPIRIT and CONSORT. Below we describe the search strategy for potentially eligible publications and the process of screening, assessing, selecting and storing them.

We seek to identify empirical studies on SPIRIT and CONSORT items and relevant studies of risk of bias, operationalized as:

1. Empirical studies of SPIRIT and CONSORT of any kind.
2. Studies comparing protocols with published randomized trials.
3. Meta-epidemiological studies of bias in randomized trials.
4. Other methodological studies of risk of bias and reporting relevant for SPIRIT-CONSORT items.

Overall considerations
Lasse Østengaard will be database coordinator while the necessary manpower for reading references and full-texts will be recruited through the network of members of the executive group.

We plan to establish the SCIP-db mainly on searches in Medline and Embase though supplemented by Web of Science searches and complementary contributions from our personal files. The ambition is to create a comprehensive database, which can be recurrently updated, but with no claim for completeness.

We will develop the preliminary database search in Embase (Ovid) because we expect that this will result in a higher number of hits compared to Medline. A very high number of papers cite SPIRIT 2013 and CONSORT 2010, which makes it impractical to look through each one. The last version of CONSORT is from 2010 and SPIRIT is from 2013. We will therefore focus on publications from 2010 and onwards. The search for studies comparing protocols with published trials will differ from the other three searches because we will use Web of Science to search for citing articles of relevant studies.

We anticipate that the number of hits for each of the four main searches will be about 20-30% higher than the Embase derived numbers displayed in the examples later in this document (after inclusion of records from Medline and removal of duplicates). However, there will also be an overlap between the four searches, so we take the sum of hits of the four Embase searches as a rough estimate of the total number of hits.

The SCIP-db is established in parallel with conducting a scoping review of comments on SPIRIT and CONSORT (see circulated protocol draft). The search strategy for the scoping review will be based on full text search, supplemented by standard database search. The broader search for the SCIP-db will primarily be based on standard databases. The overlap between the two search strategies will be adjusted by comparing the search results and removing duplicates, so one reference is screened only once.

Search no. 1 - Empirical research on SPIRIT and CONSORT

This search will consist of three parts looking for:

- Articles mentioning SPIRIT or CONSORT in the title or abstract.
- Articles about clinical trials and protocols and the quality of reporting.
- Articles about improving reporting standards.

The first part of the search will have a high sensitivity identifying articles mentioning SPIRIT or CONSORT in the title or abstract.

The last two parts of the search have a high precision since "reporting" is searched without truncation in the title/abstract. If "reporting" is searched with truncation (for example; report$) the search generates over 11.100 hits.

Embase Classic+Embase 1947 to 2020 April 17

#	Searches	Results
1	consort.ti,ab.	2980
2	consolidat$3 standard$.ti,ab.	1021
3	(SPIRIT and (protocol$ or guideline$ or statement$)).ti,ab.	657
4	Protocol$ Item$.ti,ab.	108
5	1 or 2 or 3 or 4	3886
6	((randomised or randomized or clinical or intervention$) adj3 (trial$ or study or studies)).ti,ab.	1383359
7	rct$.ti,ab.	73729
8	exp clinical trial/ or exp "clinical trial (topic)"/ or exp clinical protocol/	1882486
9	6 or 7 or 8	2570360
10	((complet$ or transparen$ or quality) adj3 reporting).ti,ab.	9720
11	9 and 10	3279
12	5 or 11	6404
13	Reporting.ti,ab.	253353
14	(standard$ adj3 (improv$ or faciltat$ or consolidat$3)).ti,ab.	16830
15	13 and 14	2049
16	12 or 15	7367
17	limit 16 to yr="2010 -Current"	5761

Search no. 2 - Studies comparing protocols with published trials

We consider the best way to ensure a reasonable balance between precision and sensitivity is to conduct the search in Web of Science. A pilot search in Embase resulted in >5000 hits.

Thus, we suggest to build the search on systematic reviews of studies comparing protocols and published trials, mainly:

1) Li, G., Abbade, L.P.F., Nwosu, I. et al. A systematic review of comparisons between protocols or registrations and full reports in primary biomedical research. BMC Med Res Methodol 18, 9 (2018).

2) Dwan, K., Altman, D. G., Cresswell, L., Blundell, M., Gamble, C. L., Williamson, P. R., & Dwan, K. (2011). Comparison of protocols and registry entries to published reports for randomised controlled trials. Cochrane Database of Systematic Reviews, 2011(1), MR000031. doi:10.1002/14651858.MR000031.pub2

Li et al. is the latest review to our knowledge. They identified 37 studies within biomedicine broadly based on a search in late 2016. We plan to:

1) Look through the 37 studies included in Li et al. to identify studies relevant for SCIP-db, i.e. those focusing on randomized trials.
2) Check that all relevant studies from Dwan et al. are included.
3) Identify publications from 2016 onwards that cite one of the SCIP-db relevant studies (see appendix for a preliminary list).

We predict this strategy will result in max 1000 hits.

Search no. 3 - Meta-epidemiological studies of bias in trials

This search is looking for:

- Meta-epidemiological studies of meta-analyses including randomized trials

This search is straightforward, and it results in a relatively low number of hits.

Embase Classic+Embase 1947 to 2020 May 22

#	Searches	Results
1	(meta-epidemiolog$ or metaepidemiolog$).ti,ab.	253
2	(Meta adj2 epidemiolog$).ti,ab.	335
3	1 or 2	351
4	limit 3 to yr="2010 -Current"	307

Search no. 4 – Reviews and methodological studies of risk of bias

This search consists of two parts looking for:

- Studies on risk of bias in randomized trials
- Methods studies relevant for randomized trials

This is the most challenging and broad search. The first part, searching for risk of bias and related terms, will generate a massive amount of hits. To have a reasonable number of hits we will only search in the title. The workload is deemed too large if this part of the search would have a high sensitivity. The second part of the search is aimed at identifying articles describing their study as a methodological study in the title. Thus, our aim is to identify central publications, but it is unavoidable and planned that we will miss some.

Embase Classic+Embase 1947 to 2020 May 22

#	Searches	Results
1	Risk of bias.ti.	368
2	((selection or performance or detection or attrition or reporting) adj1 bias).ti.	1125
3	(sequence generation or allocation concealment or incomplete outcome or selective reporting).ti.	163
4	(participant$ adj6 (retention or retaining)).ti.	170
5	(blind$ and (review or reviews or overview or overviews or survey)).ti.	943
6	(block or simple or stratified or unequal or restricted) adj1 random$.ti.	300
7	((minimi#ation or response-adaptive) and (random$ or trial$)).ti.	222
8	1 or 2 or 3 or 4 or 5 or 6 or 7	3260
9	((review or reviews or overview or overviews or study or studies or survey)).ti,ab.	14455658
10	8 and 9	2484

11	((Methodology or methodological) adj2 (review or reviews or overview or overviews or study or studies)).ti.	2558
12	10 or 11	5026
13	limit 12 to yr="2010 -Current"	2812

Workload and timing

The estimated number of references resulting from the combined search is 10.000, though this span is clearly uncertain.

Lasse will be on paternity leave until late summer. He has volunteered to take out time to conduct the primary search and distribute the lists of hits, once we have agreed on a final search strategy, and identified persons to look through the reference lists. We expect that a second supplementary round of searching will be conducted after he returns.

We have recruited five summer students and a postdoc. An-Wen and David have recruited two summer students each, as of June 1st. Sally has received positive response from postdoc Beni Speich, and Isabelle has recruited one student (maybe two). It is unclear when they can start.

Procedures and management of the references

The search for relevant studies will consist of three phases; the first title/abstract screening phase will be conducted by students, the second full text screening phase will be conducted by Beni Speich and students, and the third selection phase will be conducted by executive group members.

Lasse will conduct the searches, combine the files, delete the duplicates, and provide the students with the list of references and a link to Covidence.

First phase: Title and abstract screening

Three students will do the first screening round in Covidence.

- References will be read by one student only.
- References will be excluded if they for obvious reasons are not relevant for the update of SPIRIT/CONSORT (i.e. not related to SPIRIT/CONSORT items).
- References will be sent to full text screening if they are considered potentially relevant for the update of SPIRIT/CONSORT.

Second phase: Full text screening

Postdoc Beni Speich will conduct the full text screening with two students

- Full texts are excluded if they are not relevant for the update of SPIRIT/CONSORT.
- Full texts are included if considered relevant and tagged as
    - SPIRIT (if relevant, a note will link the references to specific SPIRIT items).
    - CONSORT (if relevant, a note will link the references to specific CONSORT items).
    - Both SPIRIT and CONSORT (if relevant, a note will link the references to specific SPIRIT/CONSORT items).
    - Comment on SPIRIT or CONSORT (this information will feed into the scoping review).

Third phase: Selection

The included and tagged full texts are divided between the members of the executive group who will confirm the relevancy of the studies. Each member of the executive group will assess a designated number of full texts based on which division principle we will agree on, for example specific SPIRIT-CONSORT items.

Lasse will create a Covidence-review for each executive group member and upload the designated full texts for assessment. The notes given in the first rounds will be transferred to the last round. The executive group member may write a note (in Covidence) if the studies are relevant to more items and any other relevant information.

Reassessment of process and workload manpower ratio
The final number of hits and full text papers to screen and assess is imprecisely estimated, and so is the time required to go through the process. We suggest informally reassessing the plan in each of the three search/assessment phases.

The reassessment could result in iterative modifications of searches (reduced sensitivity or added search terms) or running a supplementary late search. Other adjustment options are recruitment of additional hands or adjustment of working procedures.

Management of references
All included full texts will subsequently be uploaded to Zotero (or another bibliographic database accessible for all group members). If the executive group members have knowledge of any relevant studies published past 2010 they can be uploaded to a folder made for the purpose in the database. These studies will inform potential supplementary searches.

Lasse will establish a catalog of which SPIRIT/CONSORT items the studies are related to and other relevant comments provided by the executive group.

Update
We suggest that the process could be repeated every 4 months, this time involving Lasse as responsible coordinator and the executive group members as responsible for screening and selection (or for recruiting hands to do so).

Appendix

The main source of studies that compare trial protocols and corresponding published articles is: *Li, G., Abbade, L.P.F., Nwosu, I. et al. A systematic review of comparisons between protocols or registrations and full reports in primary biomedical research. BMC Med Res Methodol 18, 9 (2018).*

The study included 37 studies within biomedicine broadly, based on a search conducted in late 2016. We will include those of the 37 studies that provide information on randomized trials. We have preliminary identified the following papers:

1. Chan, A., Hróbjartsson, A., Haahr, M. T., Gøtzsche, P. C., & Altman, D. G. (2004). Empirical evidence for selective reporting of outcomes in randomized trials: Comparison of protocols to published articles. Jama, 291(20), 2457-2465. doi:10.1001/jama.291.20.2457
2. Mathieu, S., Boutron, I., Moher, D., Altman, D. G., & Ravaud, P. (2009). Comparison of registered and published primary outcomes in randomized controlled trials. Jama, 302(9), 977-984. doi:10.1001/jama.2009.1242

3. Chan, A., Krleza-Jerić, K., Schmid, I., & Altman, D. G. (2004). Outcome reporting bias in randomized trials funded by the canadian institutes of health research. CMAJ : Canadian Medical Association Journal = Journal De l'Association Medicale Canadienne, 171(7), 735-740. doi:10.1503/cmaj.1041086
4. Hannink, G., Gooszen, H. G., & Rovers, M. M. (2013). Comparison of registered and published primary outcomes in randomized clinical trials of surgical interventions. Annals of surgery, 257(5), 818–823. https://doi.org/10.1097/SLA.0b013e3182864fa3
5. Rongen, J. J., & Hannink, G. (2016). Comparison of Registered and Published Primary Outcomes in Randomized Controlled Trials of Orthopaedic Surgical Interventions. The Journal of bone and joint surgery. American volume, 98(5), 403–409. https://doi.org/10.2106/JBJS.15.00400

Appendix 2:

The full-text screening phase of the SCIP database and scoping review projects

Lasse Østengaard 24-06-2020

This note describes the full-text screening of the two projects in more detail and lists the eligibility criteria and the tags and notes for Covidence.

<u>Who will do what?</u>

Evan, Bea, Chenchen and Ariel are screening the title/abstracts (first phase) and will also screen the full-text papers (second phase).

Benjamin will do random quality checks, and provide feedback to Evan, Bea, Chenchen and Ariel if they need a second opinion. The quality checks are described later in this note.

For the SCIPdb, full-text papers are screened by a single person.

For the scoping review, full-text papers are screened by two persons. The first screener is either Evan, Bea, Chenchen or Ariel. The second screener is Camilla. Disagreements will be resolved by discussion or, if needed, arbitration by Lasse.

<u>The aim of the two projects</u>

The SCIPdb aims to identify empirical studies on SPIRIT and CONSORT items and relevant studies of risk of bias, operationalized as:

- Empirical studies of SPIRIT and CONSORT of any kind.
- Studies comparing protocols with published randomized trials.
- Meta-epidemiological studies of bias in randomized trials.
- Other methodological studies of risk of bias and reporting relevant for SPIRIT-CONSORT items.

The scoping review aims to identify and analyse:

- Comments on SPIRIT 2013 and CONSORT 2010, with special emphasis on suggestions for guideline modifications.

<u>Eligibility criteria</u>

Papers should be included if they are relevant for one or both projects. A paper is eligible if published in 2010 or later, and reports:

- An empirical study of SPIRIT and/or CONSORT
  -e.g. studies investigating the quality of reporting before and after CONSORT 2010/SPIRIT 2013.

- A comparison of protocols with published randomized trials
  -e.g. studies comparing protocols submitted to an ethical committee and subsequent publications

- A meta-epidemiological study of bias in randomized trials
  -e.g. studies investigating within a number of meta-analyses trials with and without a characteristic hypothesized to be associated with bias. A safe option is to include all studies described as a meta-epidemiological study focusing on bias and reporting in clinical trials.

- A methodological study of risk of bias and reporting relevant for SPIRIT-CONSORT items. It is challenging to a priory define what is a relevant methodological study, so if in doubt: include. A guide is that a paper should be relevant for a SPIRIT-CONSORT item (see SPIRIT 2013 and CONSORT 2010 statements), or for a potential new item (not likely, but possible). The following examples include some, but clearly not all, relevant issues:
  - Registration - trial registry.
  - Recruitment - strategies for achieving adequate participant enrollment to reach target sample size.
  - Sequence generation - method used to generate the random allocation sequence and type of randomisation; details of any restriction (such as blocking and block size).
  - Allocation concealment - mechanism used to implement the random allocation sequence (such as sequentially numbered containers).
  - Blinding - who was blinded (for example, participants, care providers, those assessing outcomes) and how.
  - Missing data - any statistical methods to handle missing data (e.g. multiple imputation).
  - Funding - sources of funding and other support (such as supply of drugs), and the role of funders.

- Comments on SPIRIT 2013 and CONSORT 2010, with special emphasis on suggestions for guideline modifications. These studies are challenging to identify as most comments on SPIRIT/CONSORT are probably not mentioned or hinted at in the abstract. For the scoping review, we expect that most included papers will be identified in a full-text search on Google scholar (conducted in parallel by Camilla), and not by the title/abstract screening you are conducting. However, if a comment is identified, either based on information in the abstract or by reading a full-text paper, it should be included. We will include opinion pieces (e.g. commentaries, letters, and editorials) as well as empirical studies and literature reviews. To be included:
  - Papers have to mention SPIRIT or CONSORT somewhere in the text (i.e. not necessarily in the title or abstract).
  - The comment on SPIRIT or CONSORT must be more than a peripheral remark (i.e. very short remarks of no more than one sentence).
  - Papers can suggest modifications to the items of at least one of the reporting guidelines, or reflect on their strengths or limitations.
  - The relevant comments will presumably be identified in commentaries, editorials or in the Discussion section of other types of papers.

Other papers are excluded.

<u>Practical hints for Covidence</u>

When you “Exclude” a full-text paper, you will be asked to provide a reason for the exclusion. Just write “not relevant”. For the SCIP database we do not list exclusion criteria. For the Scoping review, Camilla will define and organize reasons for exclusion.

Please provide tags and notes to the studies that are eligible for one or both of the projects

The tags and notes that you provide for the included publications will form the base for how they are distributed to the executive group in the third phase.

The tags

Please use one or more of the following tags for the SCIP database:

- SPIRIT
- CONSORT
- Both SPIRIT and CONSORT

Please use the following tag for the Scoping review:

- Comment on SPIRIT or CONSORT

You can combine the tags “SPIRIT”/”CONSORT”/”Both SPIRIT and CONSORT” with “Comment on SPIRIT or CONSORT”.

Notes

The notes you provide will be relevant for the SCIPdb. Please indicate the SPIRIT or CONSORT item which the publication is most relevant for.

I have added a few examples below with a *tag* for each note.

SPIRIT: ”Blinding relevant for item # X”

CONSORT: “Blinding relevant for item # X” or “randomization relevant for item # X”

Both SPIRIT and CONSORT: “Blinding relevant for item # X in SPIRIT and item # Y in CONSORT” or “comparing protocols with published randomized trials” or “meta-epidemiological study of blinding”

The quality checks

Benjamin will conduct random checks of the papers that have been screened in the second phase. The goal of this procedure is to help the screeners as much as possible by providing them with useful guidance and tips. The number of checks will be influenced by the observations that Benjamin makes. The number of screened papers will, therefore, be what Benjamin finds suitable. The checks include:

- Screening of included papers to see if they are missing a tag.
- Screening of included papers to see if the notes could be improved.
- Observation of significant differences in relation to the number of articles deemed relevant between the four screeners.
- Screening of excluded papers to see if they are relevant (for example, by conducting iterative searches in the excluded folder for relevant words such as meta-epidemiological, SPIRIT, CONSORT, etc.).

Appendix 3:

SCIPdb classification system

Background:

The development of the SPIRIT-CONSORT Informative Publications database (*SCIPdb*) started in May 2020. More than 15.000 references have been screened and the *SCIPdb* now contains 575 papers.

The next step is to decide which references that are central for the future update of the reporting guidelines. Publications that directly suggest a modification to SPIRIT or CONSORT have been identified in a parallel scoping review (headed by Camilla Hansen Nejstgaaard), and summary tables will be available in 4-6 weeks.

Aim:

To identify references relevant for SPIRIT/CONSORT or otherwise of interest:

a) references that provide an important context for the guidelines (e.g., empirical assessment of bias, adherence, new developments in planning, registration, conduct, analysis and reporting of randomised trials),

b) references that indirectly suggest a modification of SPIRIT/CONSORT, and

c) other relevant references to the expected papers.

Method:

The 575 references have been divided into six new libraries. These libraries will now be screened and tagged by one of the members of the executive group during the next six weeks. The executive group members should all have received an invitation from Zotero to their library entitled *SCIPdb – "their name"*.

When the references in the six libraries have been screened and tagged the references will be merged into one library again.

<u>Classification/tag system</u>

Level one, importance

- Tag the reference with **<u>Yes</u>** (potentially a reference relevant for SPIRIT/CONSORT or otherwise of interest)
- Tag the reference with **<u>No</u>** (most likely not a reference of particular interest)

Level two, elaboration

For all papers tagged with **<u>Yes</u>**, the assessment will be further elaborated with an additional tag(s):

- Tag the reference with one of the merged ID (1-46, max 3) e.g., **<u>ID 12</u>** (if the reference is relevant for a specific item in SPIRIT/CONSORT)
- Tag the reference with **<u>Multiple merged IDs</u>** (if the reference is relevant for more than three of the merged IDs)
- Tag the references with **<u>Empirical study of bias</u>** (if the reference is an empirical study of bias)

- Tag the reference with **<u>Comment</u>** (if the reference indirectly suggest a modification of SPIRIT/CONSORT)
- Tag the reference with **<u>Otherwise of interest</u>** (use this tag if the reference does not fit into one of the above-mentioned tags)*

*We will recommend that you write a note in Zotero describing why the reference is of interest

Plan:

The screening will take the next six weeks (from 26 October 2021 till 8 December 2021) and will be coordinated together with the scoping review.

<u>The first two weeks</u>: The members of the executive group will pilot test the screening system on ten papers and provide feedback to Asbjørn before November 9, 2021. Asbjørn and Lasse will then reassess the classification system within two working days.

<u>The last four weeks</u>: The members of the executive group will screen the remaining papers in their library.

Other information:

Distribution of the references

The references were sorted by title when they were divided into six new libraries. The libraries were distributed between the members of the executive group based on the members first names.

Comments

The scoping review headed by Camilla Hansen Nejstgaaard will summarise direct suggestions for changes or comments on SPIRIT/CONSORT but is based on screening of many of the same publications by previous screeners (i.e., student researchers). If any of the students have overlooked relevant publications, your tags with **<u>Comment</u>** will help us to identify those papers, so they can be included in the scoping review. The student researchers have used the tag **<u>Comment on SPIRIT or CONSORT</u>**, so your tag with **<u>Comment</u>** will be unique and easy to identify.

Classification system with merged IDs

	SPIRIT 2013			CONSORT 2010		
	**Section / item**	**No**	**Description**	**Section / Topic**	**No**	**Checklist item**
**MERGED ID**	**Administrative information**			**Title and abstract**		
**1**	Title	1	Descriptive title identifying the study design, population, interventions, and, if applicable, trial acronym	Title and abstract	1a	Identification as a randomised trial in the title
					1b	Structured summary of trial design, methods, results, and conclusions (for specific guidance see CONSORT for abstracts)
				**Other information**		
**2**	Trial registration	2a	Trial identifier and registry name. If not yet registered, name of intended registry	Registration	23	Registration number and name of trial registry
		2b	All items from the World Health Organization Trial Registration Data Set			
**3**	Protocol version	3	Date and version identifier			
**4**	Funding	4	Sources and types of financial, material, and other support	Funding	25	Sources of funding and other support (such as supply of drugs), role of funders
**5**	Roles and responsibilities	5a	Names, affiliations, and roles of protocol contributors			
		5b	Name and contact information for the trial sponsor			
		5c	Role of study sponsor and funders, if any, in study design; collection, management, analysis, and interpretation of data; writing of the report; and the decision to submit the report for publication, including whether they will have ultimate authority over any of these activities			

		5d	Composition, roles, and responsibilities of the coordinating centre, steering committee, endpoint adjudication committee, data management team, and other individuals or groups overseeing the trial, if applicable (see Item 21a for data monitoring committee)			
	**Introduction**			**Introduction**		
**6**	Background and rationale	6a	Description of research question and justification for undertaking the trial, including summary of relevant studies (published and unpublished) examining benefits and harms for each intervention	Background and objectives	2a	Scientific background and explanation of rationale
		6b	Explanation for choice of comparators			
**7**	Objectives	7	Specific objectives or hypotheses		2b	Specific objectives or hypotheses
				**Methods**		
**8**	Trial design	8	Description of trial design including type of trial (eg, parallel group, crossover, factorial, single group), allocation ratio, and framework (eg, superiority, equivalence, noninferiority, exploratory)	Trial design	3a	Description of trial design (such as parallel, factorial) including allocation ratio
	**Methods: Participants, interventions, and outcomes**					
**9**	Study setting	9	Description of study settings (eg, community clinic, academic hospital) and list of countries where data will be collected. Reference	Participants	4b	Settings and locations where the data were collected

			to where list of study sites can be obtained			
**10**	Eligibility criteria	10	Inclusion and exclusion criteria for participants. If applicable, eligibility criteria for study centres and individuals who will perform the interventions (eg, surgeons, psychotherapists)		4a	Eligibility criteria for participants
**11**	Interventions	11 a	Interventions for each group with sufficient detail to allow replication, including how and when they will be administered	Interventions	5	The interventions for each group with sufficient details to allow replication, including how and when they were actually administered
		11 b	Criteria for discontinuing or modifying allocated interventions for a given trial participant (eg, drug dose change in response to harms, participant request, or improving/worsening disease)			
		11 c	Strategies to improve adherence to intervention protocols, and any procedures for monitoring adherence (eg, drug tablet return, laboratory tests)			
		11 d	Relevant concomitant care and interventions that are permitted or prohibited during the trial			
**12**	Outcomes	12	Primary, secondary, and other outcomes, including the specific measurement variable (eg, systolic blood pressure), analysis	Outcomes	6a	Completely defined pre-specified primary and secondary outcome measures, including how and when they were assessed

			metric (eg, change from baseline, final value, time to event), method of aggregation (eg, median, proportion), and time point for each outcome. Explanation of the clinical relevance of chosen efficacy and harm outcomes is strongly recommended		6b	Any changes to trial outcomes after the trial commenced, with reasons
**13**	Participant timeline	13	Time schedule of enrolment, interventions (including any run-ins and washouts), assessments, and visits for participants. A schematic diagram is highly recommended (see Figure)			
**14**	Sample size	14	Estimated number of participants needed to achieve study objectives and how it was determined, including clinical and statistical assumptions supporting any sample size calculations	Sample size	7a	How sample size was determined
**15**	Recruitment	15	Strategies for achieving adequate participant enrolment to reach target sample size			
	**Methods: Assignment of interventions (for controlled trials)**					
	Allocation:			Randomisation:		
**16**	Sequence generation	16a	Method of generating the allocation sequence (eg, computer-generated random numbers), and list of any factors for stratification. To	Sequence generation	8a	Method used to generate the random allocation sequence
					8b	Type of randomisation; details of any restriction (such as

			reduce predictability of a random sequence, details of any planned restriction (eg, blocking) should be provided in a separate document that is unavailable to those who enrol participants or assign interventions			blocking and block size)
**17**	Allocation concealment mechanism	16 b	Mechanism of implementing the allocation sequence (eg, central telephone; sequentially numbered, opaque, sealed envelopes), describing any steps to conceal the sequence until interventions are assigned	Allocation concealment mechanism	9	Mechanism used to implement the random allocation sequence (such as sequentially numbered containers), describing any steps taken to conceal the sequence until interventions were assigned
**18**	Implementatio n	16 c	Who will generate the allocation sequence, who will enrol participants, and who will assign participants to interventions	Implementatio n	10	Who generated the random allocation sequence, who enrolled participants, and who assigned participants to interventions
**19**	Blinding (masking)	17 a	Who will be blinded after assignment to interventions (eg, trial participants, care providers, outcome assessors, data analysts), and how	Blinding	11 a	If done, who was blinded after assignment to interventions (for example, participants, care providers, those assessing outcomes) and how
					11 b	If relevant, description of the similarity of interventions
		17 b	If blinded, circumstances under which unblinding is permissible, and procedure for revealing a participant’s allocated intervention during the trial			

	**Methods: Data collection, management, and analysis**					
**20**	Data collection methods	18 a	Plans for assessment and collection of outcome, baseline, and other trial data, including any related processes to promote data quality (eg, duplicate measurements, training of assessors) and a description of study instruments (eg, questionnaires, laboratory tests) along with their reliability and validity, if known. Reference to where data collection forms can be found, if not in the protocol			
**21**		18 b	Plans to promote participant retention and complete follow-up, including list of any outcome data to be collected for participants who discontinue or deviate from intervention protocols			
**22**	Data management	19	Plans for data entry, coding, security, and storage, including any related processes to promote data quality (eg, double data entry; range checks for data values). Reference to where details of data management procedures can be found, if not in the protocol			
**23**	Statistical methods	20 a	Statistical methods for analysing primary and secondary outcomes. Reference to where other details	Statistical methods	12 a	Statistical methods used to compare groups for primary and secondary outcomes

			of the statistical analysis plan can be found, if not in the protocol			
		20 b	Methods for any additional analyses (eg, subgroup and adjusted analyses)		12 b	Methods for additional analyses, such as subgroup analyses and adjusted analyses
		20 c	Definition of analysis population relating to protocol non-adherence (eg, as randomised analysis), and any statistical methods to handle missing data (eg, multiple imputation)			
	**Methods: Monitoring**					
**24**	Data monitoring	21 a	Composition of data monitoring committee (DMC); summary of its role and reporting structure; statement of whether it is independent from the sponsor and competing interests; and reference to where further details about its charter can be found, if not in the protocol. Alternatively, an explanation of why a DMC is not needed	Sample size	7b	When applicable, explanation of any interim analyses and stopping guidelines
		21 b	Description of any interim analyses and stopping guidelines, including who will have access to these interim results and make the final decision to terminate the trial			
**25**	Harms	22	Plans for collecting, assessing, reporting, and managing solicited and spontaneously reported adverse events and other			

			unintended effects of trial interventions or trial conduct			
**26**	Auditing	23	Frequency and procedures for auditing trial conduct, if any, and whether the process will be independent from investigators and the sponsor			
	**Ethics and dissemination**					
**27**	Research ethics approval	24	Plans for seeking research ethics committee/institution al review board (REC/IRB) approval			
**28**	Protocol amendments	25	Plans for communicating important protocol modifications (eg, changes to eligibility criteria, outcomes, analyses) to relevant parties (eg, investigators, REC/IRBs, trial participants, trial registries, journals, regulators)	Trial design	3b	Important changes to methods after trial commencement (such as eligibility criteria), with reasons
**29**	Consent or assent	26 a	Who will obtain informed consent or assent from potential trial participants or authorised surrogates, and how (see Item 32)			
		26 b	Additional consent provisions for collection and use of participant data and biological specimens in ancillary studies, if applicable			
**30**	Confidentialit y	27	How personal information about potential and enrolled participants will be collected, shared, and maintained in order to protect confidentiality			

			before, during, and after the trial			
**31**	Declaration of interests	28	Financial and other competing interests for principal investigators for the overall trial and each study site			
**32**	Access to data	29	Statement of who will have access to the final trial dataset, and disclosure of contractual agreements that limit such access for investigators			
**33**	Ancillary and post-trial care	30	Provisions, if any, for ancillary and post-trial care, and for compensation to those who suffer harm from trial participation			
**34**	Dissemination policy	31 a	Plans for investigators and sponsor to communicate trial results to participants, healthcare professionals, the public, and other relevant groups (eg, via publication, reporting in results databases, or other data sharing arrangements), including any publication restrictions			
		31 b	Authorship eligibility guidelines and any intended use of professional writers			
		31 c	Plans, if any, for granting public access to the full protocol, participant-level dataset, and statistical code	Protocol	24	Where the full trial protocol can be accessed, if available
	**Appendices**					

**35**	Informed consent materials	32	Model consent form and other related documentation given to participants and authorised surrogates			
**36**	Biological specimens	33	Plans for collection, laboratory evaluation, and storage of biological specimens for genetic or molecular analysis in the current trial and for future use in ancillary studies, if applicable			
				**Results**		
**37**				Participant flow	13 a	For each group, the numbers of participants who were randomly assigned, received intended treatment, and were analysed for the primary outcome
					13 b	For each group, losses and exclusions after randomisation, together with reasons
**38**				Recruitment	14 a	Dates defining the periods of recruitment and follow-up
					14 b	Why the trial ended or was stopped
**39**				Baseline data	15	A table showing baseline demographic and clinical characteristics for each group
**40**				Numbers analysed	16	For each group, number of participants (denominator) included in each analysis and whether the analysis was by original assigned groups
**41**				Outcomes and estimation	17 a	For each primary and secondary outcome, results for each group, and the estimated effect size and its precision (such as 95% confidence interval)

					17 b	For binary outcomes, presentation of both absolute and relative effect sizes is recommended
**42**				Ancillary analyses	18	Results of any other analyses performed, including subgroup analyses and adjusted analyses, distinguishing pre-specified from exploratory
**43**				Harms	19	All important harms or unintended effects in each group (for specific guidance see CONSORT for harms)
				**Discussion**		
**44**				Limitations	20	Trial limitations, addressing sources of potential bias, imprecision, and, if relevant, multiplicity of analyses
**45**				Generalisability	21	Generalisability (external validity, applicability) of the trial findings
**46**				Interpretation	22	Interpretation consistent with results, balancing benefits and harms, and considering other relevant evidence

Appendix 4:

Reference list of identified topic-specific databases[a]

1. Manhã, E. M., Silva, M. C., Alves, M. G. C., Almeida, M. B., & Brandão, M. G. L. (2008). PLANT: A bibliographic database about medicinal plants. Revista Brasileira De Farmacognosia, 18(4), 614-617. https://doi.org/10.1590/S0102-695X2008000400020
2. Dilkes-Hall I, Dotte E. Pacific Matildas Bibliographic Database 2021 [Available from: https://www.zotero.org/groups/4397547/pacific_matildas_bibliographic_database accessed 4 December 2023].
3. Dozier M, Brbre I, Bissels G, Björklund M, Fry A. Review Methods Library [Available from: https://www.zotero.org/groups/2356323/review_methods_library accessed 4 December 2023].
4. Blekkenhorst, L. C., Prince, R. L., Ward, N. C., Croft, K. D., Lewis, J. R., Devine, A., Shinde, S., Woodman, R. J., Hodgson, J. M., & Bondonno, C. P. (2017). Development of a reference database for assessing dietary nitrate in vegetables. Molecular Nutrition & Food Research, 61(8). https://doi.org/10.1002/mnfr.201600982
5. Montagné-Huck, C., & Brunette, M. (2018). A bibliographic database on economic analysis of natural forest disturbances. Data in Brief, 20, 662-666. https://doi.org/10.1016/j.dib.2018.08.128
6. Geppert, C., Bogenschutz, M. P., & Miller, W. R. (2007). Development of a bibliography on religion, spirituality and addictions. Drug and Alcohol Review, 26(4), 389-395. https://doi.org/10.1080/09595230701373826
7. Hooper, L., Abdelhamid, A., Brainard, J., Deane, K. H. O., Song, F., & PUFAH group. (2019). Creation of a database to assess effects of omega-3, omega-6 and total polyunsaturated fats on health: Methodology for a set of systematic reviews. BMJ Open, 9(5), e029554-e029554. https://doi.org/10.1136/bmjopen-2019-029554
8. Gould, A. J. (1997). An introduction to the work of the acupuncture research resource centre and ARRCBASE: A bibliographic database of acupuncture practice and research. Complementary Therapies in Medicine, 5(3), 168-171. https://doi.org/10.1016/S0965-2299(97)80061-1
9. Elbing, U., Schulze, C., Zillmann, H., Raak, C. K., & Ostermann, T. (2009). Arthedata—An online database of scientific references on art therapy. European Journal of Integrative Medicine, 1(1), 39-42. https://doi.org/10.1016/j.eujim.2009.01.001
10. Hirt, J., Schönenberger, C. M., Ewald, H., Lawson, D. O., Papola, D., Rohner, R., Suter, K., Lin, S., Germini, F., Zeng, L., Shahabinezhad, A., Chowdhury, S. R., Gao, Y., Bhattacharjee, A., Lima, J. P., Marusic, A., Buljan, I., Agarwal, A., Guyatt, G. H., Briel, M., & Schandelmaier, S. (2023). Introducing the library of guidance for health scientists (LIGHTS): A living database for methods guidance. JAMA Network Open, 6(2), e2253198-e2253198. https://doi.org/10.1001/jamanetworkopen.2022.53198
11. The EQUATOR Network. [Available from: https://www.equator-network.org/ accessed 12/4 2023].
12. Rada, G., Pérez, D., Araya-Quintanilla, F., Ávila, C., Bravo-Soto, G., Bravo-Jeria, R., Cánepa, A., Capurro, D., Castro-Gutiérrez, V., Contreras, V., Edwards, J., Faúndez, J., Garrido, D., Jiménez, M., Llovet, V., Lobos, D., Madrid, F., Morel-Marambio, M., Mendoza, A., Neumann, I,. Ortiz-Muñoz, L,. Peña, J,. Pérez, M,. Pesce, F,. Rain, C,. Rivera, S,. Sepúlveda, J,. Soto, M,. Valverde, F,. Vásquez, J,. Verdugo-Paiva, F,. Vergara, C,. Zavala, C,. & Zilleruelo-Ramos, R,. on behalf of Epistemonikos project. (2020). Epistemonikos: A comprehensive database of systematic reviews for health decision-making. BMC Medical Research Methodology, 20(1), 286-286. https://doi.org/10.1186/s12874-020-01157-x
13. Herbert, R., Moseley, A., Sherrington, C. PEDro: A Database of Randomised Controlled Trials in Physiotherapy. Health Information Management 1998;28(4):186-88. https://doi.org/10.1177/183335839902800410

[a]The search for topic-specific databases was done intermittently and informally.

14. Dickersin, K., Manheimer, E., Wieland, S., Robinson, K. A., Lefebvre, C., McDonald, S., & Central Development Group. (2002). Development of the cochrane Collaboration's central register of controlled clinical trials. Evaluation & the Health Professions, 25(1), 38-64. https://doi.org/10.1177/016327870202500104
15. Badal, K., Moore, M., & Thomas, M. (2021). The repository for caribbean cancer publications (ReCCaP): Database development and publication trends 2004-2019. Cancer Management and Research, 13, 5433-5442. https://doi.org/10.2147/CMAR.S314853
16. Chua, H. H. (2005). The Avalon Project at Yale Law School: Documents in law, history and diplomacy. Emerald Group Publishing Limited. https://doi.org/10.1108/09504120510613094
17. Bernasconi, A. A., Wilkin, A. M., Roke, K., & Ismail, A. (2022). Development of a novel database to review and assess the clinical effects of EPA and DHA omega-3 fatty acids. Prostaglandins, Leukotrienes and Essential Fatty Acids, 183, 102458-102458. https://doi.org/10.1016/j.plefa.2022.102458
18. Rots AH, Winkelman S, Paltani S, et al. The Chandra bibliography database. Astronomical Data Analysis Software and Systems XIII 2004;314:605.
19. Barth, J., Wang, J., Lopez-Alcalde, J., Kramm, C., Pach, D., Álvarez-Díaz, N., Grifol-Clar, E., & Witt, C. M. (2022). Smartphone-RCCT: An online repository of randomized controlled clinical trials of smartphone applications for chronic conditions. Current Controlled Trials in Cardiovascular Medicine, 23(1), 909-909. https://doi.org/10.1186/s13063-022-06849-x20. Livingston, K. A., Chung, M., Sawicki, C. M., Lyle, B. J., Wang, D. D., Roberts, S. B., & McKeown, N. M. (2016). Development of a publicly available, comprehensive database of fiber and health outcomes: Rationale and methods. PloS One, 11(6), e0156961-e0156961. https://doi.org/10.1371/journal.pone.015696121. Kearney, A., Harman, N. L., Rosala-Hallas, A., Beecher, C., Blazeby, J. M., Bower, P., Clarke, M., Cragg, W., Duane, S., Gardner, H., Healy, P., Maguire, L., Mills, N., Rooshenas, L., Rowlands, C., Treweek, S., Vellinga, A., Williamson, P. R., & Gamble, C. (2018). Development of an online resource for recruitment research in clinical trials to organise and map current literature. Clinical Trials (London, England), 15(6), 533-542. https://doi.org/10.1177/1740774518796156
22. O'Neil, M. E., Harik, J. M., McDonagh, M. S., Cheney, T. P., Hsu, F. C., Cameron, D. C., Carlson, K. F., Norman, S. B., & Hamblen, J. L. (2020). Development of the PTSD-Repository: A publicly available repository of randomized controlled trials for posttraumatic stress disorder. Journal of Traumatic Stress, 33(4), 410-419. https://doi.org/10.1002/jts.22520
23. JUSTNORTH. Justice Database 2022 [Available from: https://justnorth.eu/outcome/database/ accessed December 20, 2022.
24. Priya, P., Patil, M., Pandey, P., Singh, A., Babu, V. S., & Senthil-Kumar, M. (2023). Stress combinations and their interactions in plants database: A one-stop resource on combined stress responses in plants. The Plant Journal : For Cell and Molecular Biology, 116(4), 1097-1117. https://doi.org/10.1111/tpj.16497
25. Latitudes Network [Available from: https://www.latitudes-network.org/ accessed 12/04 2023].
